# NEUROPSYCHIATRIC SYMPTOMS OF DEMENTIA AND CAREGIVERS’ BURDEN

**DOI:** 10.1093/geroni/igad104.2412

**Published:** 2023-12-21

**Authors:** Constanca Paul, Susana Sousa, Laetitia Teixeira

**Affiliations:** ICBAS, Portugal, Porto, Porto, Portugal; ICBAS, University of Porto, Porto, Porto, Portugal; School of Medicine and Biomedical Sciences, University of Porto, Porto, Porto, Portugal

## Abstract

Informal caregiving (IC) is a major health and social asset although simultaneously a potential burden and depression risk factor for those who care. Neuropsychiatric symptoms of dementia are disturbing and may contribute to a negative experience of care. To understand the neuropsychiatric symptoms of dementia in caregiving we have two objectives: to know the prevalence of neuropsychiatric symptoms perceived by ICs and if there is an association of neuropsychiatric symptoms and burden and depression of caregiver. We study a community-based sample (N=101) of primary care users with mental health concerns, referred by GPs. Instruments include sociodemographic information; Neuropsychiatric Inventory (NPI-Q), Caregiver Burden Scale (CBS) and Geriatric Depression Scale (ABCDS). The sample mean age was 79.4 (sd=7.7) years, 54 (53.5%) were female and 34 (33.7%) had no formal education. Half of the sample were married and 41 (41.90%) were widowed. The NPI-Q total score and the Distress dimension score were 10.1 (sd=5.6) and 11.9 (sd=9.1), respectively. The more reported symptoms were agitation/aggression (69.3%), and apathy/indifference (65.3%). We found a significant positive association between NPI-Q and caregivers’ burden (0.296) and depression (0.227) and between Distress and caregivers’ burden (0.417) and depression, 0.416. Conclusion: Neuropsychiatric symptoms of dementia appear associated with the burden and depression of informal caregivers. Training ICs to cope with care receiver symptoms is a priority to increase the quality of care and reduce burden and depression of caregivers.

